# A high-throughput protocol for testing heat-stress tolerance in pollen

**DOI:** 10.1007/s42994-024-00183-3

**Published:** 2024-10-14

**Authors:** Chenchen Zhao, Abu Bakar Siddique, Ce Guo, Sergey Shabala, Chengdao Li, Zhonghua Chen, Rajeev Varshney, Meixue Zhou

**Affiliations:** 1 Tasmanian Institute of Agriculture, University of Tasmania, Prospect, TAS 7250 Australia; 2 International Research Centre for Environmental Membrane Biology, Foshan University, Foshan, 528000 China; 3 School of Biological Science, University of Western Australia, Crawley, 6009 Australia; 4 Food Futures Institute, School of Agriculture, Western Crop Genetics Alliance, Murdoch University, Murdoch, WA 6150 Australia; 5 Agriculture and Food, Department of Primary Industries and Regional Development, South Perth, WA 6150 Australia; 6 School of Science, Western Sydney University, Penrith, NSW 2751 Australia; 7 Hawkesbury Institute for the Environment, Western Sydney University, Penrith, 2751 Australia

**Keywords:** Heat stress, Pollen germination, Pollen tube development, *Triticum aestivum* L., *Hordeum vulgare* L.

## Abstract

Viable pollen is crucial for fertilization, but pollen is generally highly susceptible to heat stress. A quick, reliable method for testing the heat-stress tolerance of pollen is needed to improve the heat-stress tolerance in plants, but current methods require considerable space and labor. In addition, many such methods only test tolerance to a single constant temperature, making it time-consuming to screen heat tolerance over a wide temperature range and to examine the dynamics of pollen viability at different temperatures. To address this issue, we aimed to: (1) develop an easy, reliable method for measuring pollen viability at different temperatures; and (2) identify the best temperature range for screening pollen with high heat-stress tolerance. We harvested mature pollen from wheat (*Triticum aestivum*) plants and transferred it to a 96-well plate filled with liquid medium containing sucrose. We placed the plate in a PCR machine operating under a gradient PCR program to simultaneously test a range of temperatures. After incubating the pollen for 4 h, at temperatures ranging from 21.9 to 47 °C, we examined the pollen grains under a light microscope and employed a specific image analysis pipeline to assess the effects of temperature on pollen morphology, germination, and tube growth. This method facilitated the high-throughput screening of many pollen samples, enabling rapid, reliable, and precise analysis of pollen viability in response to temperature. Our approach should be applicable to other plant species and could be used to identify quantitative trait loci or genes influencing heat stress tolerance in pollen for breeding programs.

**Supplementary Information:**

The online version contains supplementary material available at 10.1007/s42994-024-00183-3.

## Introduction

During vegetative growth, plants may experience temperature stress when outside their optimal temperature range by 5–10 °C. Plant reproductive growth is more sensitive to heat stress than vegetative growth (Zinn et al. 2010; Gao et al. 2022). In particular, pollen development requires a narrower temperature range than other developmental stages, as its complex developmental program requires the coordinated activity of different gametophytic and sporophytic cell types and tissues (Hafidh et al. 2016). This makes pollen development susceptible to temperature stress, especially heat stress (Bheemanahalli et al. 2019; Khan et al. 2019; Thakur et al. 2010), which causes aberrant pollen formation, diminished pollen tube growth, and defects in tropism, resulting in decreased fertility (Zinn et al. 2010). Since pollen viability and function are crucial for crop yields, identifying plant varieties that produce pollen with enhanced heat-stress tolerance has important implications for crop breeding.

Wheat (*Triticum aestivum* L.) has distinct optimal temperature ranges for reproductive and vegetative growth (Farooq et al. 2011). High temperatures (heat stress) detrimentally affect pollen germination and pollen tube growth in wheat (Asseng et al. 2015; Stratonovitch and Semenov 2015; Tashiro and Wardlaw 1990), resulting in morphological abnormalities and decreased fertilization and seed yield. Even brief episodes of heat stress (daily temperatures exceeding 24 °C) during the heading stage decrease floret fertility, with floret fertility and individual grain weight exhibiting a linear decline along with increasing duration of heat stress from 2 to 30 days (Prasad and Djanaguiraman 2014).

Reactive oxygen species (ROS) play key roles in normal pollen development and in the metabolic reactions associated with heat-induced decreases in pollen fertility. The tapetum is a layer of nutritive cells that provide nutrition for the developing pollen grains. ROS-induced programmed cell death (PCD) of tapetum cells at the proper time releases pollen grains for fertilization. However, under heat stress, tapetum cells accumulate ROS, which triggers premature PCD and can result in male sterility (Kurusu and Kuchitsu 2017). In addition to their effects on tapetal cells, ROS also directly damage pollen; for example, excess ROS damaged the pollen membrane, resulting in collapsed and desiccated pollen and ultimately a lower seed-setting rate in sorghum (*Sorghum bicolor*) (Djanaguiraman et al. 2018). Heat stress induces ROS accumulation in anthers and disrupts the balance of ROS-scavenging enzymes. Moreover, warm temperatures lead to the increased production of antioxidant compounds such as flavonoids and polyamines (Paupière et al. 2017), thus directly affecting ROS homeostasis (Chen et al. 2019; Rice-Evans et al. 1996). Collectively, the effects of heat stress can lead to premature PCD and degradation of the tapetal cell layer (Zhao et al. 2018).

Substantial research has examined the effects of heat stress on pollen germination, pollen tube growth, and pollen viability in various crop species, including wheat (Bheemanahalli et al. 2019), barley (*Hordeum vulgare*) (Burke et al. 2004; Impe et al. 2020), cotton (*Gossypium hirsutum*) (Burke et al. 2004), maize (*Zea mays*) (Bheemanahalli et al. 2022), rice (*Oryza sativa*) (Rang et al. 2011), and others (Domínguez et al. 2005; Jiang et al. 2017). However, most of these studies have focused on a single temperature condition. Some studies have employed growth chambers set to diverse temperature regimens, but these setups typically take several days to complete the heat treatment (Jiang et al. 2015), require substantial amounts of space, and are affected by variables such as plant height and growth chamber size (Porch and Jahn 2001). Efficiently examining the effects of temperature stress on pollen viability and identifying the optimal temperature for screening heat-tolerant wheat varieties require a reliable, rapid screening method.

In this study, we developed a rapid, efficient protocol using gradient PCR programs to investigate the effects of a range of temperatures on pollen germination and pollen tube growth. We used gradient PCR programs to test temperatures from 21.9 to 47.0 °C, thus covering a broad range of temperatures spanning from optimal to extreme temperatures. Following incubation in the PCR machine, we examined the pollen germination rate and pollen growth rate under a stereomicroscope and quantified the results using an image analysis pipeline, enabling us to translate visual data into quantitative data. To validate this approach, we investigated the response of pollen viability in barley to heat exposure using the same protocol. We also chose nine wheat cultivars with contrasting heat tolerance phenotypes to investigate the possible correlation between pollen viability and seed-setting rate under heat stress.

## Results

### Development and setup of the assay to test a wide range of temperatures for heat stress

**Fig. 1:**
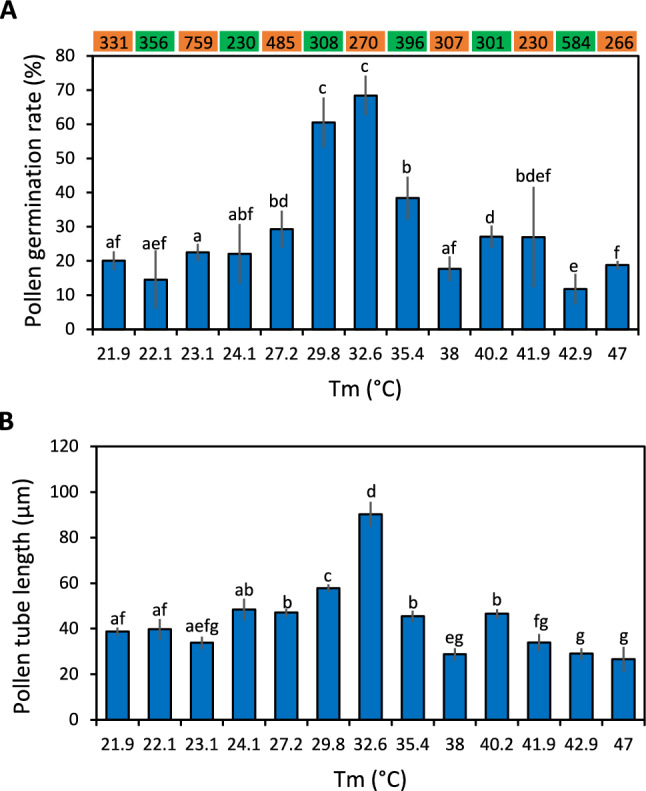
Assessment of wheat pollen germination rate and pollen tube length as a function of temperature. **A** Pollen germination rate and **B** changes in pollen tube length processed from images of stained pollen grains for each temperature regime using ImageJ. Three views under a microscope were processed, with over 200 individual pollen grains investigated per temperature. Values are means ± standard error (SE, *n* = 4). The colored boxes above the plots indicate the number of pollen grains investigated. Different lowercase letters represent statistically significant differences (one-way ANOVA, *P* < 0.05)

**Fig. 2:**
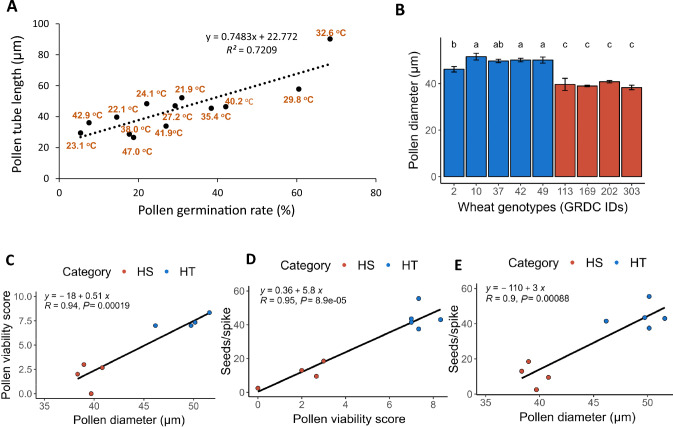
Correlation analysis between pollen germination rate and various aspects of pollen morphology. **A** Scatterplot showing the relationship between pollen tube length and pollen germination rate. **B** Pollen diameter for the heat-tolerant (blue) and heat-sensitive (red) wheat genotypes tested in this study. **C–E** Scatterplots showing the relationship between pollen diameter and pollen viability (**C**), pollen viability and spike fertility as determined by the number of seeds per spike (**D**), and pollen diameter and spike fertility (**E**). For each temperature regime, three views under a microscope were processed, with over 200 individual pollen grains investigated. Each data point is the average value from these three views

The protocol we developed uses a PCR machine to expose different pollen grain samples to various temperatures simultaneously, then visualizes the pollen using an inverted microscope and converts the visual data into quantified data using image processing software. This protocol facilitates the comprehensive study of the effects of different temperatures on pollen germination and pollen tube growth. The PCR machine functions as an incubator, producing accurate, precise temperatures over a broad range, in theory allowing the user to investigate the effects of temperatures from 0 to 95 °C on pollen viability (Fig. S1). It is important to note that the achievable temperature range depends on the capabilities of the available PCR machine, but most PCR machines can maintain consistent temperatures within this range. This system also enables prolonged pollen incubation under variable temperature regimens, ranging from a few minutes to several hours in duration, by adjusting the program settings. In the dark environment of the PCR machine, the pollen grains still germinated and responded to heat stress. Using this protocol, we measured the germination rate and tube length of viable pollen with mature pollen exposed to different temperatures showing significant differences in their germination rates and tube lengths (Figs. 1, 2).

### Assessing pollen germination rates and pollen tube growth at different temperatures

**Fig. 3:**
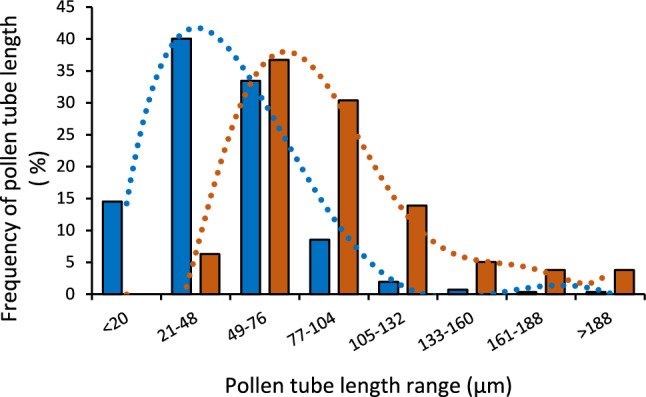
Distribution of pollen tube length from the investigated pollen grains. The lengths of pollen tubes measured under all temperature regimes except the optimal temperature (32.6 °C) are shown in blue, whereas the lengths of pollen tubes measured under the optimal temperature of 32.6 °C are shown in orange

**Fig. 4:**
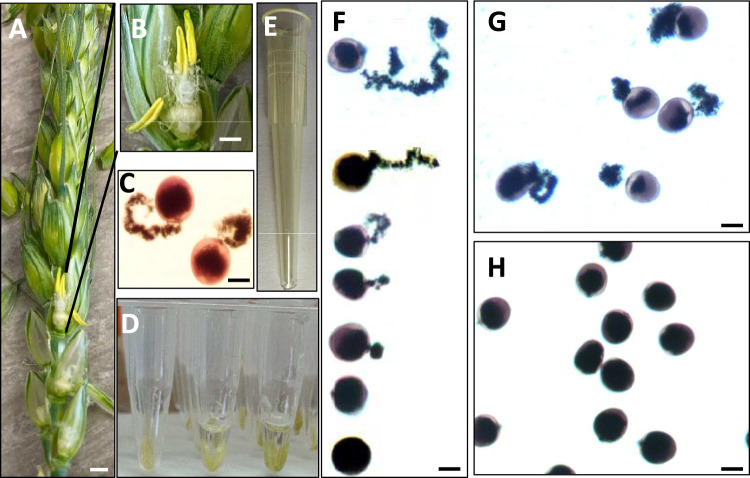
Representative pollen sampling time points and pollen performance under variable temperature conditions. At the flowering stage when mature anthers turn yellow and become dehydrated (**A**, **B**), round, healthy pollen grains (**C**) were collected for investigation. Three anthers were collected and incubated in each well of a 96-well plate filled with 20 µL of pollen culture medium (**D**). After heat incubation, a yellow pipette tip with the end cut off (**E**) was used to gently resuspend the incubated pollen grains for examination under a microscope. **F** Various examples of pollen tubes after incubating pollen grains at different temperatures ranging from low (23.1 °C) to optimal (32.6 °C). **G** Pollen germination following incubation at the optimal temperature. **H** Pollen germination following incubation at room temperature (~ 23 °C). Scale bars in (**A**, **B**), 20 mm; in (**C**, **F**–**H**), 20 µm

We observed an increase in the pollen germination rate and pollen tube length when the temperature increased from 21.9 to 32.6 °C (Fig. 1). The pollen germination rate exceeded 60% at temperatures from 29.8 to 32.6 °C (Fig. 1A). When the temperature increased to 35.4 °C, the germination rate dropped to less than 40%, and the average pollen tube length declined to less than 49.6% of the length measured at the optimal temperature of 32.6 °C. These results indicate that a temperature of 32.6 °C is optimal for pollen germination and pollen tube growth in the pollen culture medium used in this study (Fig. 1). The length of pollen tubes followed a normal distribution, with the optimal temperature resulting in more germinated pollen grains with longer tubes than any other temperature tested (Fig. 3). Whereas mature, healthy pollen grains were round, with a good germination rate at the optimal temperature (Fig. 4), various other temperature treatments ranging from below optimum to optimal temperatures (23–32.6 °C) yielded diverse pollen tube lengths (Fig. 4F). Incubating pollen grains at the optimal temperature led to substantially enhanced pollen tube elongation (top of Fig. 4F) and germination rates (Fig. 4G). Pollen grains incubated at room temperature showed less germination within the short (4 h) period used for the assay (Fig. 4H), which is consistent with the results shown in Figs. 1 and 2.

We used the same method to test the viability of mature pollen grains collected from barley plants (Fig. S2). Pollen germination rates exhibited a trend similar to those of wheat pollen, but with a slightly different optimal temperature of 27.2 °C (Fig. S2), which is consistent with the lower heat tolerance of barley compared with wheat (https://blog-crop-news.extension.umn.edu/2021/06/heat-stress-on-small-grains.html).

### Correlation between pollen germination rate and pollen tube growth

Successful seed set is contingent upon pollen grain germination and the elongation of the pollen tube to reach the ovule (Chakrabarti et al. 2011). We determined that the pollen germination rate is closely and positively correlated with the length of pollen tubes (*R*^2^ = 0.72, Fig. 2A). We observed the lowest pollen germination rate and the shortest pollen tubes for pollen incubated at temperatures below 23.1 °C and above 47 °C, with germination rates of 5.4 and 18.7% and average pollen tube lengths of 29.5 µm and 26.6 µm, respectively (Fig. 2A).

### Correlation between pollen grain sizes and pollen viability as well as spikelet fertility under heat stress

The size of pollen grains is positively correlated with pollen viability in yellow monkeyflower (*Mimulus guttatus*) and blue-eyed Mary (*Collinsia verna*) (Kelly et al. 2002). Here, we investigated pollen size for nine wheat cultivars grown in the field and explored the correlation between pollen viability under heat stress and pollen size. We selected the nine genotypes for their contrasting heat tolerance phenotypes. Genotypes that were more tolerant of heat stress (as indicated by their higher seed-setting rate under heat stress in the growth room, Table 1) had significantly larger pollen grains than susceptible genotypes (Fig. 2B). We also detected a significant positive correlation between pollen size and pollen viability (Fig. 2C), with larger pollen grains more viable upon exposure to heat stress than smaller pollen grains. Not surprisingly, both pollen viability and pollen size were significantly and positively correlated with spikelet fertility under heat stress (Fig. 2D, E).

**Table 1:** Spikelet fertility and pollen performance under heat stress for nine selected wheat cultivars

Wheat genotype	Heat-stress tolerance	Average pollen viability score (0–10)	Average pollen diameter (µm)	Seed number per 100 spikelets	Seed weight per spike (g)	Seed number per spike
GRDC002	HT	7.3 ± 0.6	46.2 ± 1.1	236.8 ± 11.0	1.01 ± 0.10	41.5 ± 2.0
GRDC010	HT	7.7 ± 0.6	51.6 ± 1.4	253.1 ± 12.6	0.98 ± 0.04	43.0 ± 2.1
GRDC037	HT	7.9 ± 0.8	49.7 ± 0.7	228.95 ± 11.0	1.02 ± 0.03	43.5 ± 2.1
GRDC042	HT	7.1 ± 0.8	50.1 ± 0.8	226.1 ± 10.9	0.79 ± 0.03	37.5 ± 1.9
GRDC049	HT	7.6 ± 0.4	50.1 ± 1.3	258.2 ± 12.9	0.89 ± 0.04	55.5 ± 2.7
GRDC113	HS	1.6 ± 1.8	39.6 ± 2.6	11.4 ± 0.6	0.05 ± 0.00	2.5 ± 0.1
GRDC169	HS	2.7 ± 0.3	39.0 ± 0.3	88.1 ± 4.4	0.57 ± 0.03	18.5 ± 1.0
GRDC202	HS	2.4 ± 1.0	40.8 ± 0.5	45.2 ± 2.3	0.12 ± 0.00	9.5 ± 0.5
GRDC303	HS	2.0 ± 0.0	38.3 ± 1.0	70.2 ± 3.5	0.49 ± 0.03	13.0 ± 0.7

*HT* heat-tolerant, *HS* heat-sensitive

## Discussion

Pollen viability serves as a comprehensive indicator of pollen performance, encompassing fertilization capability and the ability to germinate (Dafni and Firmage 2000). Pollen viability is commonly estimated through in vitro germination assays, allowing pollen tube growth to be analyzed in liquid or solid medium using essentially the same method developed decades ago (Brewbaker and Kwack 1963). Subsequent studies have modified the culture medium to optimize pollen performance: for example, a later study on wheat pollen grains achieved outstanding tube lengths of up to 200 µm even after only a 1-h incubation using a medium containing raffinose (Cheng and McComb 1992). However, due to the high cost of raffinose, other studies that replaced raffinose with sucrose or maltose resulted in pollen germination rates of up to 95% (Jayaprakash et al. 2015). Therefore, we chose a sucrose-containing medium in the current study.

Pollen viability is vulnerable to heat stress, making it a key trait for selecting and breeding heat-tolerant crop varieties. In a field trial, late sowing caused plants to be exposed to temperatures over 30 °C during the reproductive stage, which significantly decreased grain yield and resulted in crop losses (Harohalli Masthigowda et al. 2022). In the current study, 32.6 °C was the optimal temperature at which we achieved the greatest germination rate and tube growth of wheat pollen in sucrose-containing culture medium, which is slightly different from the field-based findings of Harohalli Masthigowda et al. (2022). Because heat stress is commonly accompanied by ambient drought in the field, this might have created more pronounced temperature stress on pollen in the field. By contrast, the sucrose-containing culture medium used in this study did not impose drought or osmotic stress, which may explain the higher optimal temperature range obtained here. Heat stress also diminishes pollen tube growth, preventing the pollen from fertilizing the egg and central cell, thus resulting in spikelet sterility, as observed in rice (Shi et al. 2018). These findings closely align with our results, as high temperatures inhibited pollen tube growth and caused a low pollen germination rate (Figs. 1, 2A). The underlying mechanism may involve the production of ROS, which is strongly induced by heat stress.

Most previous studies on the consequences of temperature stress on pollen viability were time-consuming and demanded large experimental spaces to accommodate the growth of many plants under varying temperature regimes (Begcy et al. 2018; Browne et al. 2021; Shenoda et al. 2021). In this study, we developed an innovative protocol for monitoring the activity of wheat pollen grains, allowing us to utilize temperatures ranging from 22 to 40 °C, covering 13 temperature settings (Fig. S1). In contrast to previous studies, in which several days of incubation were typically needed before yielding results (Burke et al. 2004; Chaturvedi et al. 2021; Impe et al. 2020), the protocol we developed requires only a few hours of incubation for pollen germination and pollen tube elongation, confirming that a brief incubation period can be sufficient for pollen germination and pollen tube growth (Burke et al. 2004). This new screening protocol enables the simultaneous testing of 96 genotypes at a specific temperature, making it well-suited for mapping quantitative trait loci (QTLs) for heat-stress tolerance in pollen or for direct use in breeding programs. The protocol also provides optimal conditions for identifying the temperature requirements for both pollen germination and pollen tube growth (Fig. 1).

We validated our new method using mature barley pollen grains. We identified a lower temperature threshold for heat-stress tolerance in barley compared to wheat (Fig. S2), which is consistent with a previous report. We observed a significant positive correlation between pollen grain size and pollen viability under heat stress conditions (Fig. 2), as reported previously (Kelly et al. 2002). Larger pollen grains often exhibit higher viability (Kelly et al. 2002; Bonciu 2013), most likely due to their greater levels of carbohydrate reserves (Firon et al. 2006), which enhance their germination potential and pollen tube growth under heat stress. A method was previously developed to estimate pollen viability based on size variation, highlighting the importance of this trait for reproductive success (Ayenan et al. 2020). Here, an analysis of genotypes selected for their differing levels of heat-stress tolerance indicated that the heat-stress tolerance of pollen is significantly correlated with spikelet fertility under heat-stress conditions (Fig. 2D, E), highlighting the usefulness of our method for breeding wheat varieties for heat-stress tolerance.

In conclusion, we present a flexible, convenient, and efficient screening protocol for analyzing the responses of pollen to temperature stress (Figs. 1, 3). This protocol enables the simultaneous assessment of pollen germination and pollen tube growth under varying temperatures within a short time frame, ranging from minutes to a few hours. With the capacity to accommodate up to 12 different temperatures in the same assay, this protocol facilitates the identification of the optimal temperature for pollen performance. Additionally, the protocol is integrated with a streamlined image recording and analysis procedure, allowing comprehensive analysis of various morphological traits in pollen, including grain size, viability, and pollen tube length across different varieties. Further experiments are needed to examine the expression of heat shock–related genes in pollen before and after heat-stress treatment.

## Materials and methods

### Plant materials and experimental design

Ten wheat varieties (nine listed in Table 1 and wheat cultivar ‘TP13501’) and one barley cultivar (‘Planet’) were used in this study. The nine varieties listed in Table 1 were selected from over 300 genotypes tested in a glasshouse trial, with five varieties consistently presenting higher pollen viability and four varieties showing low pollen viability (Table 1). To validate the relationship between the heat tolerance of pollen and spikelet fertility under high-temperature conditions, these nine wheat genotypes were sown in pots filled with potting mixture (P2POTT, Sustainable Timber Tasmania, TAS, 7250). When the plants reached the anthesis stage in the glasshouse, the pots were transferred to a growth room under a 16-h light/8-h dark photoperiod with 800 PAR (photosynthetically active radiation) and a day/night temperature regime of 35–42 °C/25–30 °C for heat-stress treatment (Bheemanahalli et al. 2019). After maturity, the seed-setting rate and number of seeds per spike were recorded for each genotype.

### Plant growth, pollen selection, and sample preparation

All 300 wheat genotypes and barley cultivar planet were sown in mid-September 2023 in the Tasmanian Institute of Agriculture field site at Mount Pleasant Laboratories, Launceston, TAS, Australia (41.47°S, 147.14°E). The seeds were planted in 0.6-m rows with 15 seeds per row. The subsequent field maintenance adhered to local farmers’ practices. During the first floret opening time (mid-December), the main spike and three primary spikes were selected for screening method development (Fig. S3A and B). The selected anthers exhibited a fresh yellow color, indicating that the pollen was viable and mature (Fig. S3C). Mature pollen grains were carefully collected using tweezers and transferred into the wells of a 96-well plate, with each well filled with 20 µL of pollen liquid culture medium (Harohalli Masthigowda et al. 2022). Mature, healthy anthers, typically characterized by their sinking to the bottom of the plate, released their pollen grains into the liquid culture medium (Fig. 2D, Fig. S3D). The anthers were placed in a 96-well plate (Sigma Aldrich, USA), a lid was placed on the plate, and the plate was subjected to gentle vibration (~ 120 rpm) using a vortex mixer (RATEK, Vortex Shaker, Australia) for 30 s before being loaded into the PCR machine (Eppendorf 5331 Mastercycler Gradient Thermal Cycle, USA) under the gradient PCR program defined in this study (Fig. S1A). A total of 12 gradient temperatures (Fig. S1B) were tested. For each temperature, three anthers from one spike were collected and incubated in one plate well, with four spikes from four independent plants serving as four biological replicates. The anther selection and sample preparation procedures followed Phase 1 of the protocol shown in Fig. S3.

### Gradient PCR protocol

The configuration of the PCR program closely resembled a default gradient PCR procedure with some modifications (Fig. S1C). Specifically, the lid temperature of the PCR machine was set to 25 °C, and the volume of liquid medium in the 96-well plate for pollen incubation was set to 20 µL (comprising pollen germination medium and anthers). In accordance with previous findings on the heat-stress tolerance of wheat pollen, a temperature range from 22 to 40 °C was selected for this investigation (Fig. S1C), where ‘T’ denotes the medium temperature and ‘G’ denotes the gradient temperatures (Fig. S1C). The entire program, as displayed on the PCR machine, is illustrated in Fig. S1C. The incubation period was set to 4 h.

### Pollen germination and pollen growth rate

After incubation in the PCR machine with the above gradient PCR protocol, the narrow end of a yellow pipette tip was cut off, and the tip was extended to the bottom of the plate wells and used to gently resuspend the pollen grains (Fig. 4E). Subsequently, 10 µL of culture medium containing the treated pollen grains was carefully transferred to a glass slide. To check pollen viability, an additional 10 µL of 1% (w/v) potassium iodide (KI) was added to the slide. The slide was gently spun by hand to concentrate the pollen grains toward the center of the slides to overcome electrostatic effects. Images of stained pollen grains were captured using an upright microscope under a 10 × 10 scope (Olympus BH2, fitted with an OMAX A35180U3 fixed microscope camera) equipped with ToupView for image digitalization. Pollen germination rate and pollen tube length were assessed using ImageJ (Abràmoff et al. 2004). The methods for calculating germination rate, assessing pollen status after incubation at different temperatures, and the specifics of data processing are provided in Phase 2 of the protocol, as shown in Fig. S3.

### Statistical analysis

All statistical analyses were conducted using R software (Version 4.1.2). ANOVA was employed for side-by-side comparisons under the assumptions of normal distribution and homogeneity of variances. Curve fitting using polynomial correlation was applied to analyze the normal distributions of pollen germination and pollen tube elongation. Linear correlation through curve fitting was implemented to examine the relationship between pollen germination rate and pollen tube elongation, as well as between temperatures and pollen grain size.

## Supplementary Information

Below is the link to the electronic supplementary material.

Supplementary file1 (DOCX 948 kb)

## Data Availability

All data generated or obtained and analyzed in this study are included in this published article and the documents provided in supplementary information.
