# Supplementary material for: A high-throughput protocol for testing heat-stress tolerance in pollen
Source: aBIOTECH. 2024 Oct 14;6(1):63–71. doi: 10.1007/s42994-024-00183-3 (PMC11889306; doi:10.1007/s42994-024-00183-3)
Supplement: Supplementary file 1 — Supplementary file1 (DOCX 948 kb) [file 42994_2024_183_MOESM1_ESM.docx]

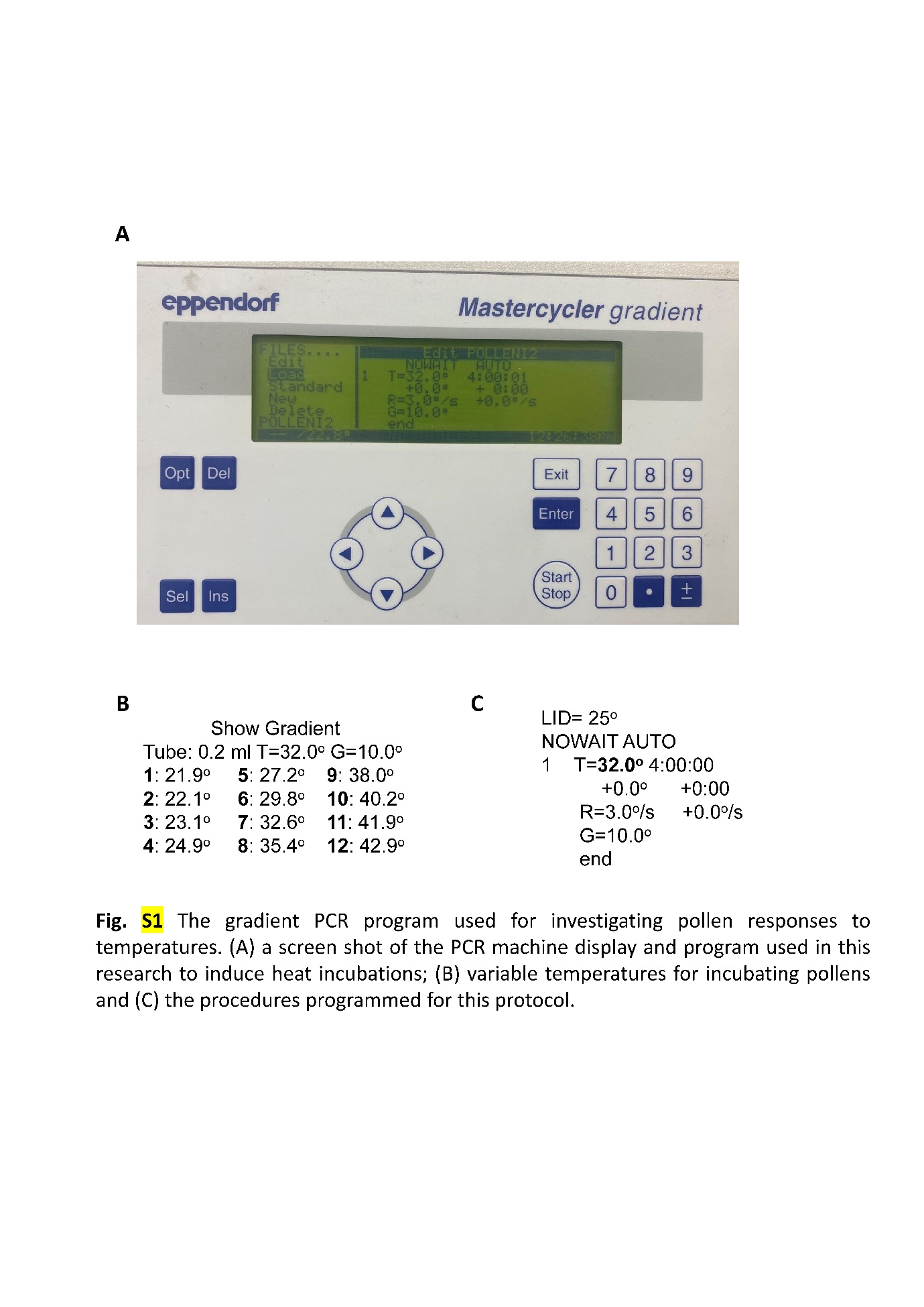


**Fig. S1** The gradient PCR program used for investigating pollen responses to temperatures. (**A**) a screen shot of the PCR machine display and program used in this research to induce heat incubations; (**B**) variable temperatures for incubating pollens and (**C**) the procedures programmed for this protocol.


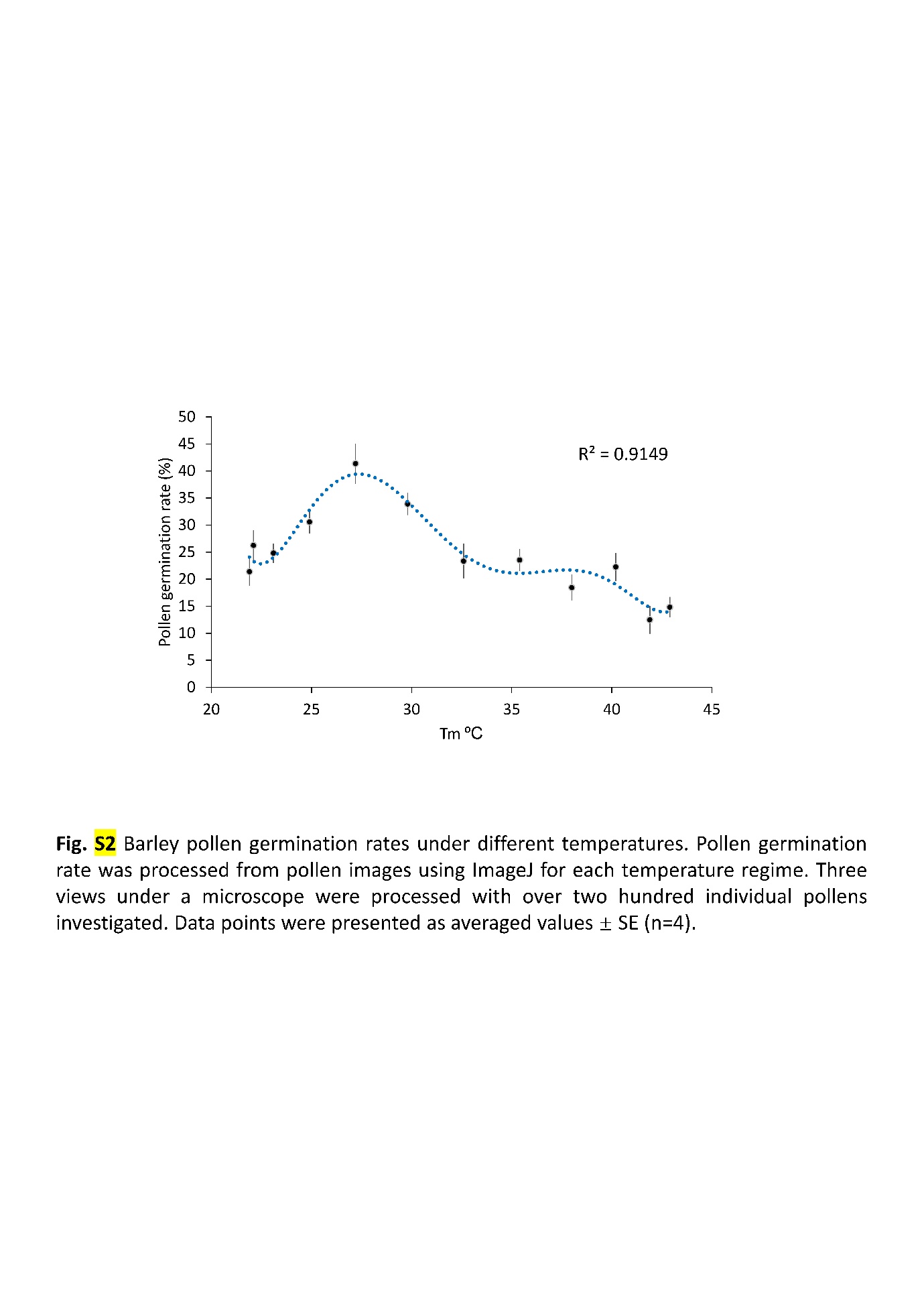


**Fig. S2** Barley pollen germination rate under different temperatures. Pollen germination rate was processed from pollen images using Image J for each temperature regime. Three views under a microscope were processed with over two hundred individual pollens investigated. Data points were presented as averaged values ± SE (*n* = 4).


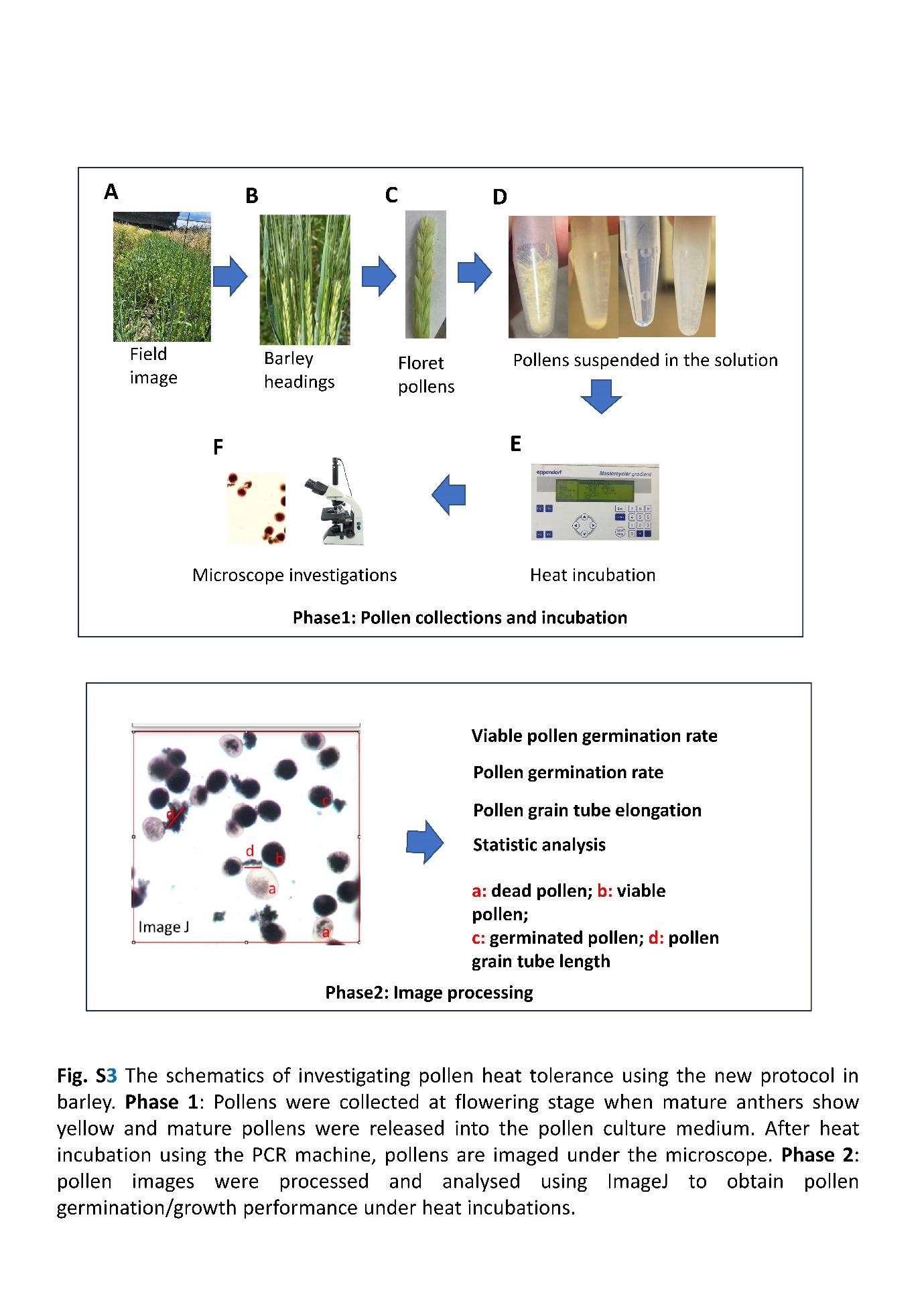


**Fig. S3** The schematics of investigating pollen heat tolerance using the new protocol in barley. Phase 1: Pollens were collected at flowering stage when mature anthers show yellow and mature pollens were released into the pollen culture medium. After heat incubation using the PCR machine, pollens are imaged under the microscope. Phase 2: pollen images were processed and analysed using ImageJ to obtain pollen germination/growth performance under heat incubations.
